# An ecological measure to screen executive functioning in MS: the Picture Interpretation Test (PIT) 360°

**DOI:** 10.1038/s41598-019-42201-1

**Published:** 2019-04-05

**Authors:** Olivia Realdon, Silvia Serino, Federica Savazzi, Federica Rossetto, Pietro Cipresso, Thomas D. Parsons, Giacomo Cappellini, Fabrizia Mantovani, Laura Mendozzi, Raffaello Nemni, Giuseppe Riva, Francesca Baglio

**Affiliations:** 1 Università degli Studi di Milano-Bicocca, Department of Human Sciences for Education, Piazza Ateneo Nuovo 1, 20126 Milan, Italy; 2 Università Cattolica del Sacro Cuore, Department of Psychology, Largo Gemelli 1, 20123 Milan, Italy; 3 IRCCS Istituto Auxologico Italiano, Applied Technology for Neuro-Psychology LAB, via Magnasco 2, 20149 Milan, Italy; 4 IRCCS, Fondazione Don Carlo Gnocchi, Neurorehabilitation Unit and Imaging in Rehabilitation LAB, Via Capecelatro, 66, 20148 Milan, Italy; 5 University of North Texas, Computational Neuropsychology and Simulation Laboratory, 1155 Union Circle #311280, Denton, Texas 76203-5017 USA; 6 National Research Council of Italy, Institute for the Dynamics of Environmental Processes, Piazza della Scienza, 1, 20126 Milan, Italy; 7 IRCCS, Fondazione Don Carlo Gnocchi, Multiple Sclerosis Unit, Via Capecelatro, 66, 20148 Milan, Italy; 8 IRCCS, Fondazione Don Carlo Gnocchi, Neurorehabilitation Unit, Via Capecelatro, 66, 20148 Milan, Italy; 9 Università degli Studi di Milano, Department of Pathophysiology and Transplantation, Via Francesco Sforza 35, 20122 Milan, Italy

## Abstract

Executive functions are crucial for performance of everyday activities. In Multiple Sclerosis (MS), executive dysfunctions can be apparent from the early onset of the disease. Technology-based time-efficient and resource-saving tools for early evaluation of executive functions using an ecological approach are needed to assess functional performance in real-life. The aim was to compare the efficiency of the Picture Interpretation Test 360° (PIT 360°) with traditional measures on executive dysfunction in Persons with Multiple Sclerosis (PwMS) and Healthy Controls (HC). Participants were 31 patients with Relapsing-Remitting MS (mean age = 44.323 ± 13.149; mean Expanded Disability Status Scale = 2) and 39 HC (mean age = 39.538 ± 15.728). All were tested with standard neuropsychological tests of executive functions, PIT 360°, and measures of user experience. While standard neuropsychological tests failed to differentiate between PwMS and HC group, the PIT 360° was successful in detecting executive dysfunction in PwMS. All participants reported the PIT 360° to be an engaging tool and endorsed positive reactions to their experience. Overall, the PIT 360° is a quick, sensitive, and ecological tool that captures real-world executive dysfunction in PwMS. This engaging measure is sensitive for the detection of executive deficits since the early phases of the disease.

## Introduction

Cognitive impairment in Multiple Sclerosis (MS) includes, among other deficits, executive dysfunction, multitasking difficulties, verbal fluency declines, and visuo-spatial deficits^1–3^. Cognitive impairment has been found in all disease subtypes^4^, including one-third of patients with early Relapsing-Remitting (RR) MS^5^. Early onset of difficulties in simultaneous management of everyday activities is often reported in Persons with Multiple Sclerosis (PwMS)^6^. Altogether, these impairments have a disruptive impact on quality of life and the ability of PwMS to actively adapt to the changing demands of the physical and social environment^7,8^. Although conventional neuropsychological tests exist for assessing cognitive dysfunction in PwMS, they tend to be limited in their capacity for capturing the sorts of executive functioning deficits that are critical for functioning in real-world contexts^6^.

Evaluating functional performance across a range of real-life situations is the core of the function-led approach to the assessment of executive functions^9^. As highlighted by Chan and colleagues^10^, this approach, rather than fractionating the executive dysfunction, aims to incorporate the complexity of real-life challenges into tasks able to tap a number of executive domains simultaneously. Using this approach, executive functions can be captured in a context-specific scenario that enhances prediction of everyday functional behaviours. Along this line, Rouaud and colleagues^11^ showed that ecological tests could detect executive dysfunction in PwMS that was underestimated by conventional neuropsychological assessments. In an update on strategies for assessing cognition in PwMS, Ruet and Brochet^12^ pointed out that neuropsychological and ecological tests are indeed complementary tools for assessing cognitive dysfunction in everyday-like conditions.

Several tools for assessing executive functioning using an ecological approach have been developed through the application of novel technologies like Virtual Reality (VR). VR platforms allow for the development of ecologically valid assessment that simulates everyday activities in secure scenarios^13^. Virtual environments for the assessment of cognitive impairments have already been developed and empirically validated with regard to several clinical conditions^14–16^, including MS, as in the Urban DailyCog task^17^.

It is widely recognized that conventional paper and pencil tests for the assessment of cognitive status in MS are time- and resource- consuming, limiting their incorporation in standard MS care^18^. There is a wide consensus on the need for short and well validated tools that can be seamlessly incorporated in everyday clinical practice^18^. Sumowski and colleagues^6^ therefore advocated the development and validation of technology-enhanced brief and resource-efficient tools – be they computer- or tablet-based – as a key priority in measuring cognitive impairments in this population.

With the PIT 360°^19^ we advance a quick and ecological tool for the evaluation of dysexecutive deficits. The PIT 360° - which is a 360° version of the Picture Interpretation Test^20,21^ based on Luria and colleagues’ work^22^ - proved to be effective in the screening of executive dysfunction in Parkinson’s Disease (PD) in comparison to healthy controls.

In the present study we apply the PIT 360° to the evaluation of executive dysfunction in PwMS. We predicted that PIT 360° would be able to capture real-world executive dysfunction, in PwMS, in a quick and more sensitive way than conventional assessment tests.

## Results

### Participants’ characteristics and conventional neuropsychological assessment of executive functions

Table 1 exhibits results from the comparison between groups (PwMS vs Healthy Control, HC) on baseline characteristics and neuropsychological assessment scores. No significant difference was detected between the two groups for gender [*χ*^2^ = 0.695; df = 1, *p* = 0.405], age [t(67,804) = 1.386, *p* = 0.170], or education [t(68) = −1.869, *p* = 0.066]. Findings obtained from Independent Student’s t-tests indicated no significant differences between PwMS and HC group with respect to the three sub-tests of TMT [TMT-A, t(68) = −1.048; TMT-B, t(68) = −0.213; TMT-BA, t(68) = 0.073] or Verbal Fluency [t(68) = −1.209]. When compared with HC, PwMS obtained a significantly lower MoCA score [t (54,730) = −2.906].

**Table 1:** Sociodemographics, neuropsychological assessment and PIT 360° scores for PwMS and HC groups.

	PwMS group [N = 31]	HC group [N = 39]	Group comparison^a^ *p*_value
Age(years) [mean ± SD]	44.323 ± 13.149	39.538 ± 15.728	0.170
Education (years) [mean ± SD]	15.161 ± 2.922	16.385 ± 2.551	0.066
Gender (males:females)	15:16	15:24	0.405
EDSS [mean ± SD]	2.350 ± 1.750	—	—
Disease Duration (years) [mean ± SD]	12.387 ± 9.583	—	—
MoCA [mean ± SD]	24.902 ± 1.971	26.145 ± 1.497	0.005^b^
TMT-A [mean ± SD]	32.871 ± 12.099	36.154 ± 13.698	0.298
TMT-B [mean ± SD]	98.032 ± 36.892	100.128 ± 43.808	0.832
TMT-BA [mean ± SD]	64.258 ± 27.922	63.718 ± 33.114	0.942
Verbal Fluency (FAS form) [mean ± SD]	35.229 ± 10.479	38.259 ± 10.361	0.231
PIT 360°, time [mean ± SD]	65.249 ± 55.307	33.048 ± 19.565	0.024
PIT 360°, number [mean ± SD]	8.355 ± 7.153	3.872 ± 3.299	0.063

Abbreviations: PwMS, People with Multiple Sclerosis; HC, Healthy Control; EDSS, Expanded Disability Status Scale; MoCA, Montreal Cognitive Assessment; TMT, Trail Making Test; FAS, administration letters to test Verbal Fluency; PIT, Picture Interpretation Test.

^a^Sociodemographic and neuropsychological variables were compared at baseline using t test or *χ*^2^ as appropriate.

^b^Because the Levene’s test was significant, the t-test reported assumes that variances were not equal and df = 54.730.

### Performance on PIT 360°

Results in both indices obtained from the PIT 360° (see Table 1) revealed greater performance deficits for PwMS group when compared to the HC group. The PwMS group took more time (mean = 65.249; SD = 55.307) when compared to the HC group (mean = 33.048; SD = 19.565) for interpreting the PIT 360° scene [F(1,66) = 5.329; *p* = 0.024; Partial η^2^ = 0.075]. Moreover, PwMS reported a higher number of scene elements in comparison to the HC group [MS, mean = 8.355; SD = 7.153; HC, mean = 3.872, SD = 3.299; F(1,34) = 4.449; *p* = 0.039; Partial η^2^ = 0.063].

### User experience assessment

Findings from comparison of the number of self-reported felt emotions and their intensities (in each of the four Geneva Emotion Wheel - GEW - quadrants) between the two groups (PwMS vs. HC), revealed a significant difference in the number of emotions with negative valence and high coping potential. In this specific quadrant, in comparison to PwMS group, the HC group reported a significantly higher number of self-reported emotions.

No significant differences were observed between groups with respect to the overall number of self-reported felt emotions and their intensities (Table 2).

**Table 2:** Scores obtained from the user experience assessment for PwMS and HC. Data are shown as Means (M) and Standard Deviations (SD).

Scale	Subscale		PwMS Group [M (SD)]	HC Group [M (SD)]	Z	*p*	N^a^
GEW	GEW 1*- Positive Valence/High coping potential*						
	Number of emotions		2.806 (1.1667)	2.842 (1.326)	−0.137	0.891	68
	Intensity		3.433 (0.685)	3.373 (1.092)	−0.043	0.965	68
	GEW 2 *– Positive Valence/Low coping potential*						
	Number of emotions		1.000 (0.856)	1.184 (1.159)	−0.470	0.639	68
	Intensity		2.561 (0.972)	2.856 (0.960)	−0.867	0.386	47
	GEW 3 – *Negative Valence/Low coping potential*						
	Number of emotions		0.097 (0.396)	0.263 (0.601)	−1.444	0.149	68
	Intensity		1.500 (0.707)	1.357 (0.475)	−0.327	0.743	10
	GEW 4 *– Negative Valence/High coping potential*						
	Number of emotions		0.064 (0.359)	0.526 (1.109)	−2.375	0.018	68
	Intensity		3.00	2.713 (0.985)	/	/	
FSS	Perceived coping skills		3.548 (0.850)	3.769 (0.667)	−1.291	0.197	70
	Perceived challenge		2.548 (0.624)	2.103 (0.598)	−2.873	0.004	70
	Perceived challenge-skill balance		2.323 (0.600)	2.179 (0.644)	−0.914	0.361	70
IMI			3.006 (0.881)	3.369 (0.810)	−1.790	0.078	70
SUS Questionnaire			3.667 (1.510)	3.863 (1.675)	−0.509	0.612	70

Abbreviations: PwMS group, People with Multiple Sclerosis; HC group, Healthy Control; M, Mean; SD, Standard Deviation; GEW, Geneva Emotion Wheel; FSS, Flow Short Scale; IMI, Intrinsic Motivation Inventory; SUS, Slater-Usoh-Steed.

^a^One participant in the “HC group” was excluded from the analyses of GEW data because of problems in data collection.

However, findings obtained from the Friedman Test revealed a significant difference among the four quadrants of GEW in terms of the number of self-reported emotions [*χ*^2^(3) = 155.285; *p* < 0.001].

Wilcoxon tests (with Bonferroni adjustment) indicated that all participants experienced a higher number of emotions with positive valence and high coping potential (Table 3).

**Table 3:** Results obtained from Wilcoxon Test comparisons of different quadrants in the Geneva Emotion Wheel (GEW) - Mean number of self reported emotions (SD).

			U	*p*
**GEW 1**	3.400 (0.923)	vs. GEW2	−6.711	<0.001
		vs. GEW3	−7.181	<0.001
		vs. GEW4	−6.934	<0.001
**GEW 2**	2.718 (0.967)	vs. GEW3	−5.89	<0.001
		vs. GEW4	−4.793	<0.001
**GEW 3**		vs. GEW4	−1.317	0.241
	1.389 (0.486)			
**GEW 4**				
	2.742 (0.989)			

Assessment using the Flow Short Scale (FSS) revealed that the PwMS group perceived a higher level of challenges when confronted with the proposed activity in comparison to HC. However, no significant between-group difference emerged with respect to the perceived level of skills and challenge-skills balance (Table 2).

Both groups endorsed a high appreciation for the activity (Intrinsic Motivation Inventory - IMI) and an intense sense of presence (Slater-Usoh-Steed - SUS - Questionnaire) (Table 2).

### Classification of PwMS or HC

Tables 4 and 5 show the classification results for discriminating between the HC and the PwMS groups. Naïve Bayes and Support Vector Machine algorithms emerged as the best algorithms for classifying HC and PwMS in their respective groups. Using the scores from conventional executive functions tests as input, the machine learning algorithms showed a classification accuracy between 52.9% and 65.7%. In contrast, the indices from PIT 360° achieved a higher classification accuracy, ranging from 65.7% to 72.9%.

**Table 4:** Stratified 10-fold Cross validation for the neuropsychological assessment battery.

Method	AUC	CA	F1	Precision	Recall
Logistic Regression	0.494	0.529	0.653	0.554	0.795
Random Forest	0.627	0.643	0.706	0.652	0.769
Support Vector Machine (SVM)	0.636	0.657	0.727	0.653	0.821
Naïve Bayes	0.617	0.629	0.683	0.651	0.718

^a^AUC (Area under the ROC curve) is the area under the classic receiver-operating curve. CA (Classification accuracy) represents the proportion of the examples that were classified correctly; F1 represents the weighted harmonic average of the precision and recall (defined below); Precision represents a proportion of true positives among all the instances classified as positive. In our case, the proportion of a condition was identified correctly; Recall represents the proportion of true positives among the positive instances in our data.

**Table 5:** Stratified 10-fold Cross validation for the indexes of PIT360°.

Method	AUC	CA	F1	Precision	Recall
Logistic Regression	0.639	0.657	0.721	0.660	0.795
Random Forest	0.707	0.729	0.787	0.700	0.897
Support Vector Machine (SVM)	0.668	0.700	0.779	0.661	0.949
Naïve Bayes	0.678	0.700	0.764	0.680	0.872

^a^AUC (Area under the ROC curve) is the area under the classic receiver-operating curve. CA (Classification accuracy) represents the proportion of the examples that were classified correctly; F1 represents the weighted harmonic average of the precision and recall (defined below); Precision represents a proportion of true positives among all the instances classified as positive. In our case, the proportion of a condition was identified correctly; Recall represents the proportion of true positives among the positive instances in our data.

**Figure 1:**
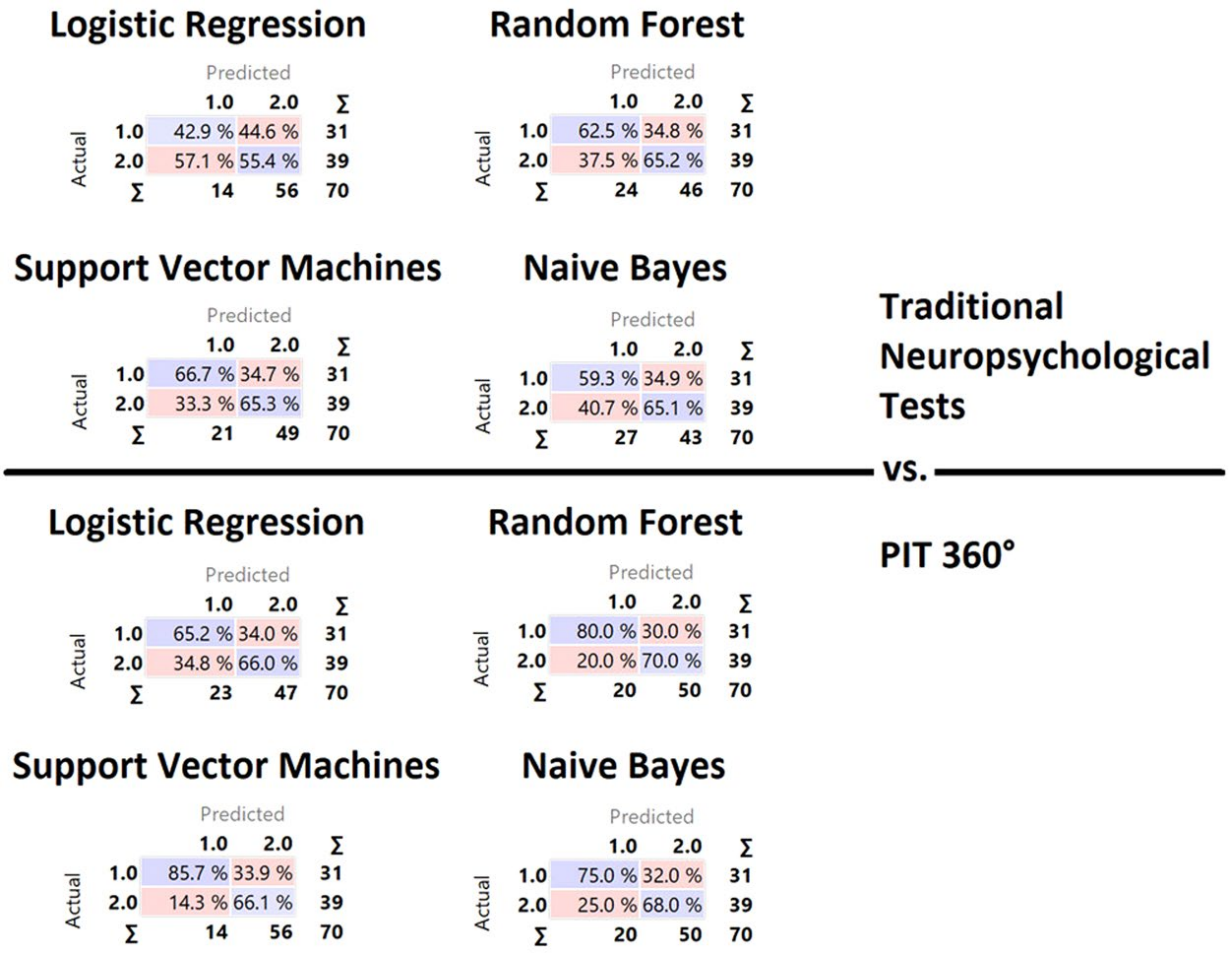
Confusion matrix. The confusion matrix of all classifiers computed for the classification into “HC Group” and “PwMS Group”. The values on the diagonal (i.e., purple values) indicate the elements for which the predicted group is equal to the true group, while off-diagonal values are those that are mislabelled by the classifiers. Results showed that PIT 360° had a higher capability for correctly classifying PwMS in their group with respect to traditional neuropsychological tests of executive functioning.

Figure 1 shows the confusion matrix of all classifiers used for classifying individuals into PwMS Group and HC Group. Results revealed that indices from the PIT 360° had a higher capability for correctly classifying PwMS in their group.

## Discussion

We aimed to evaluate the efficacy of a 360° version of the PIT for detecting executive dysfunction in PwMS through a function-led approach that combined experimental control with a real-world engaging background. In line with research findings on MS that reveal cognitive impairments that can be characterized as executive dysfunction^6^, the PwMS performed significantly worse on the PIT 360° than did persons in the HC group.

While the mean global cognitive level of PwMS in our study was lower than that of HC, it was still in a non-pathological range. This suggests initial subclinical global dysfunction in PwMS. This initial dysfunction detected with a renowned test sensitive to MS-related cognitive impairment is in line with the prior work detecting cognitive impairment in PwMS^23^. It is important to note that although verbal fluency is a sensitive tool for assessing executive impairment in PwMS and is part of the minimal assessment of cognitive function in MS (MACFIMS battery)^24^, the assessment with this test and the TMT failed in showing differences in executive functions between groups.

Different from standard neuropsychological tests used, the PIT 360° differentiated successfully between the pathological and the control conditions both in terms of time to give an answer and in number of elements in the scene. This result showed that PIT 360° is an ecological tool that is highly sensitive to MS pathology—even in its initial phases (EDSS, range 1–3). These findings were also confirmed by the higher accuracy in the Random Forest classification of participants to the clinical or non-clinical conditions (when using indices from PIT 360°) with respect to those from neuropsychological assessment. These robust findings demonstrated the efficacy of PIT 360° for detecting impairment of executive functions at an early clinical stage of MS. Moreover, they suggest that this ecological tool can be used for prompt diagnosis and early enrollment of PwMS in targeted rehabilitation^4^. The importance of an early management of cognitive impairment in MS is highlighted by the fact that it can predate the onset of physical disability and slow cognitive decline^7,25^.

Although the findings of the present study are promising, in the comparison to standard neuropsychological assessment, PIT 360° is only a very sensitive screening tool not covering the need for a full and analytical examination of executive functions. In addition, it is a technology-based test implying the use of a VR headset with potential side-effects (e.g. nausea) in some patients.

Considering findings related to users’ experiences, we found that PIT 360° was considered to be an engaging tool both by the HC and the PwMS groups. Firstly, all participants reported a good sense of presence in the 360° scene (SUS Questionnaire) showing that they actively experienced the task in a context perceived as a real-life place. Both groups rated the challenge of the PIT 360° task as feasible, in the sense that it was considered balanced with respect to their skills (FSS scale). Furthermore, PwMS and HC positively assessed participant appreciation for and interest in performing PIT 360° task (IMI scale).

All participants endorsed positive reactions to the task, showing that their experience of the PIT 360° was highly pleasant and under control. This was apparent in the higher number of self reported emotions in the first quadrant of the GEW, which includes interest, joy, happiness, satisfaction, elation and pride. Interestingly, HC vs. PwMS reported a higher number of self-reported emotions with high coping potential and negative valence. This finding can be related to the higher level of challenges perceived by the PwMS group when faced with the proposed activity in comparison to HC (FSS scale). A possible interpretation of these results is that PwMS exerted greater attentional effort when attempting to complete the task which, most probably, was higher than that of HC.

Interestingly, the efficiency of PIT 360° in detecting executive dysfunctioning was observed also in PD^19^ but was lower compared to SM. This is not surprising because executive function disorders are defined in functional terms and not as a topographic syndrome^26^ and are assessed by functional-led approach in PIT 360°. The difference in the accuracy of classification in the two clinical conditions with respect to HC may be due to several reasons. The aging can represent a factor: people with PD were older than PwMS for the natural history of the disease. Moreover, it is well known that aging is associated with decline in executive function and in the two studies were included different HC groups due to the age-related demographics of the two neurological conditions. Then, it is reasonable to expect the different sensitivity in classification accuracy in a middle-aged vs. older adults sample. Furthermore, the overall brain profile vary in the two conditions involving fronto-subcortical degeneration in PD and white matter frontal pathway disconnection in MS. Therefore, the degree of severity of the brain damage can impact executive functioning differently in the two diseases.

Finally, PIT 360° offers a promise for answering the need for time-efficient and resource-saving tools that can screen PwMS for executive deficits. This reduces patient stress at the first evaluation and orients clinicians to perform subsequent clinical investigations using longer neuropsychological assessment batteries and the prompt inclusion in targeted rehabilitation programs.

Future studies should examine PIT 360° efficacy in detecting executive dysfunctioning with a larger cohort and with other clinical populations. Moreover, it will be important that the PIT 360° be investigated using neuroimaging to establish neural correlates. Additionally, it will be of major importance to proceed with the validation of PIT 360° parallel forms to make possible a short-term re-evaluation of executive functions.

In conclusion, the PIT 360° is a quick and ecological measure that demonstrated effective and sensitive screening of real-world deficits related to executive functioning in the early stages of MS. These findings support, within Parsons’ theoretical framework^13^ for the assessment of executive functions, the methodological note advanced by Sumowski and colleagues^6^ on the need of advancing effective, evidence-based, clinically feasible understanding and measurement of dysexecutive functioning.

## Materials and Methods

### Participants

Seventy participants took part in the study: thirty-one PwMS (51.6% female; mean age = 44.323 ± 13.149; mean years of education = 15.161 ± 2.922; PwMS group) and thirty-nine healthy controls (61.5% female; mean age = 39.538 ± 15.728; mean years of education = 16.385 ± 2.551; HC group).

Outpatients meeting the diagnostic criteria for clinically definite MS^27^ with a RR disease course were consecutively recruited from the MS Unit of Don Carlo Gnocchi Foundation, IRCCS. All patients were at a mild stage of the disease, scoring between 1 and 3 of the Expanded Disability Status Scale (EDSS).

Exclusion criteria were as follows: less than 6 months from diagnosis, documented relapses within the last 3 months, severe psychiatric and neurological disorders other than MS.

The study was conducted in accordance with the Helsinki Declaration of 1975, as revised in 2013 and approved by the Local Ethics Committee (IRCCS Don Carlo Gnocchi Foundation). Written informed consent was obtained for all participants before study initiation.

### Procedure of the study and measures

The study was carried out in three subsequent steps, as in Serino and colleagues^21^. After conventional neuropsychological assessment, we administered the PIT 360° session. Next, we evaluated participants’ experiences relative to their subjective feelings, intrinsic motivations, balance between resources and demands while performing the task. Additionally, their sense of presence in the 360° environment was assessed.

#### Pre-task evaluation: neuropsychological measures

We used the same battery as in Serino et colleagues^19^: global cognitive level was assessed with the Montreal Cognitive Assessment (MoCA)^28^ – which has been shown to be sensitive in identifying MS-related cognitive impairment^29^; executive functioning was assessed using the Trail Making Test^30^ (in two specific sub-tests: TMT-A and TMT-B) as a visuo-spatial examination with an index of time; PIT 360°; and a measure of phonemic verbal fluency, the controlled oral word association test (FAS form)^23^.

#### PIT 360° session

**Figure 2:**
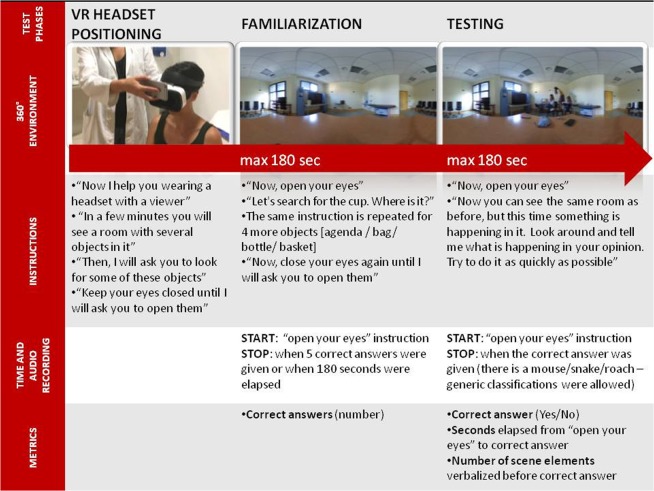
PIT 360° session. *In case of presbyopia, participants were asked to wear their own glasses. **The familiarization phase also allowed control for potential side effects (e.g., dizziness, nausea). The examiner followed a cessation rule in which experimental sessions should be stopped if severe side effects occurred. ***All persons depicted in the pictures were experimenters and signed an informed consent for publication of identifying images in an online open-access publication.

The PIT 360°^19^ is the 360° version of the Picture Interpretation Test (PIT)^20,21^. The PIT 360° environment consists of a scene in a contemporary real-world room with three frightened girls standing on chairs and a boy who is searching for something on the floor. Although not visible, it is apparent that there is a mouse (or some other small animal) hidden behind a piece of furniture. This scene is a present-day adaptation of the painting “Il Sorcio” (“The Mouse”, 1878, by Giacomo Favretto). Participants undergo a visual exploration task in which they are asked to interpret what is happening in a limited time frame. Time to correct interpretation of the scene (“There is a mouse/small animal”) and number of scene elements before correct interpretation are the outcome metrics. Session components and their unfolding over time are illustrated in Fig. 2.

#### Post-task evaluation: user experience assessment

After task completion, we evaluated a) self-reported subjective feelings through the Geneva Emotion Wheel (GEW)^31^. This tool provides a wheel shaped arrangement of 20 emotion words. Emotion labels are considered as indices “reflecting a unique experience of mental and bodily changes in the context of being confronted with a particular event”^32^. The wheel is displayed on a space formed by the underlying dimensions of valence (negative to positive) and control/coping potential (low to high). The orthogonal combination of these dimensions generates four quadrants: negative valence - low control; negative valence - high control; positive valence - low control; positive valence - high control. Subjective feelings about performing the task were rated through the mean number of emotion labels (range 0–5) and the respective reported intensity (range 1–5) within each quadrant; b) we also evaluated the skill-demands compatibility through the Flow Short Scale (FSS)^33,34^ assessing the perceived level of skills in coping with the task (“Perceived coping skills”), the perceived level of challenges of the task (“Perceived challenge”), and the perceived challenge-skill balance (“Perceived challenge- skill balance”) on a 5-points Likert scale; c) intrinsic motivation in performing the task was measured using the Interest/Enjoyment subscale of the Intrinsic Motivation Inventory (IMI; Deci)^35^. The mean of the item scores (N = 5, 7-points Likert scale) was considered; d) finally, we measured the sense of presence experienced in the 360° environment through the Slater-Usoh-Steed Questionnaire (SUS Questionnaire)^36^. The scale evaluated participants’ sense of being present in the 360° scene, and the extent to which experiencing the scene using the PIT 360° became the dominant reality and recall as a place, through three items on 7-point scale.

### Data analyses

First, the Kolmogorov-Smirnov test was used to check for the normality of data distribution for all the variables. Independent Student’s t-tests and chi-square tests were used to compare group baseline characteristics. Then, independent Student’s t-tests were carried out to explore between-group differences in the conventional assessment of executive functions (i.e., MoCA, TMT and phonemic fluency task). Two univariate analyses of variance with age and education as covariates (ANCOVA) were used to investigate PIT 360° differences in performances between HC group and PwMS group on the two performance indices (i.e., Correct Interpretation and Number of Scene Element). Since the distribution of these two variables differed moderately from normal, a square root transformation was tried. With this procedure, data were closer to the normal distribution as assessed with the Kolmogorov-Smirnov test.

Next, differences in conventional tests of executive functions between the two groups were evaluated using non-parametric tests (Wilcoxon tests). A univariate analysis of covariance (ANCOVA) with age and education as covariates was carried out to investigate differences between HC and PwMS groups in the indexes of PIT 360° (i.e., Correct Interpretation and Number of Scene Element). To investigate potential differences between the HC group and the PwMS group in user experience variables (i.e., GEW, FSS, IMI, and SUS Questionnaire), we performed independent Student’s t-tests (for normal variables) and Mann-Whitney U tests (for not normal variables). As specifically concerns the number of self-reported emotions, the Friedman test was used to explore differences within the four quadrants of the GEW. A series of Bonferroni adjusted Wilcoxon tests were subsequently computed to explore significant effects. All these statistical analyses were conducted using the Statistical Package for the Social Sciences for Windows (SPSS Inc., Chicago, IL, USA), version 23.

To compare the classification accuracy of traditional tests of executive functions and indices from PIT 360°, nonlinear stochastic approximation (i.e., machine learning) methods were employed. In particular, a leave-one-out cross-validation was carried out with the following methods (as in our previous study)^19^: (a) a Logistic Regression classification algorithm with ridge regularization; (b) a Random Forest classification to classify features using an ensemble of decision trees; (c) a Support Vector Machine (SVM) to map inputs to higher-dimensional feature spaces that best separate different features; (d) a naïve Bayes classification. All these analyses were computed using Python 3.4 with the Orange 3.3.5 data mining suite, which was available free in open source code (https://github.com/biolab/orange3).

## Data Availability

The datasets generated during and/or analysed during the current study are available from the corresponding author on reasonable request.
